# Sex Differences in Comorbidity, Therapy, and Health Services’ Use of Heart Failure in Spain: Evidence from Real-World Data

**DOI:** 10.3390/ijerph17062136

**Published:** 2020-03-23

**Authors:** Anyuli Gracia Gutiérrez, Beatriz Poblador-Plou, Alexandra Prados-Torres, Fernando J Ruiz Laiglesia, Antonio Gimeno-Miguel

**Affiliations:** 1 Research Group on Heart Failure, IIS Aragón, Internal Medicine Service, Hospital General de la Defensa, 50009 Zaragoza, Spain; agraciagut@gmail.com; 2 EpiChron Research Group, Aragon Health Sciences Institute (IACS), IIS Aragón, REDISSEC, Miguel Servet University Hospital, 50009 Zaragoza, Spain; bpoblador.iacs@aragon.es (B.P.-P.); sprados.iacs@aragon.es (A.P.-T.); 3 Research Group on Heart Failure, Faculty of Medicine, Internal Medicine Service, Lozano Blesa University Hospital, 50009 Zaragoza, Spain; fruizl@unizar.es

**Keywords:** Heart failure, epidemiology, comorbidity, medication, health services use, sex, gender

## Abstract

Heart failure (HF) is becoming increasingly prevalent and affects both men and women. However, women have traditionally been underrepresented in HF clinical trials. In this study, we aimed to analyze sex differences in the comorbidity, therapy, and health services’ use of HF patients. We conducted a cross-sectional study in Aragón (Spain) and described the characteristics of 17,516 patients with HF. Women were more frequent (57.4 vs. 42.6%, *p* < 0.001) and older (83 vs. 80 years, *p* < 0.001) than men, and presented a 33% lower risk of 1-year mortality (*p* < 0.001). Both sexes showed similar disease burdens, and 80% suffered six or more diseases. Some comorbidities were clearly sex-specific, such as arthritis, depression, and hypothyroidism in women, and arrhythmias, ischemic heart disease, and COPD in men. Men were more frequently anti-aggregated and anti-coagulated and received more angiotensin-converting-enzyme (ACE) inhibitors and beta-blockers, whereas women had more angiotensin II antagonists, antiinflammatories, antidepressants, and thyroid hormones dispensed. Men were admitted to specialists (79.0 vs. 70.6%, *p* < 0.001), hospital (47.0 vs. 38.1%, *p* < 0.001), and emergency services (57.6 vs. 52.7%, *p* < 0.001) more frequently than women. Our results highlight the need to conduct future studies to confirm the existence of these differences and of developing separate HF management guidelines for men and women that take into account their sex-specific comorbidity.

## 1. Introduction

Heart failure (HF) is a complex clinical syndrome characterized by the reduced ability of the heart to pump and/or fill with blood [1]. This condition is becoming increasingly prevalent and currently affects around 26 million people, challenging health systems worldwide [2].

Sex differences in the epidemiology, comorbidity, pharmacological treatment, and management of HF have been highlighted [3,4,5,6,7,8,9]. Although HF incidence is initially higher in men, women survive longer after the onset of the disease and, as one’s age advances, its prevalence becomes higher in women compared to men [8]. Subsequently, the total number of patients affected by HF in the general population is similar in both sexes, or even higher in women [7]. Yet, women have traditionally been underrepresented in HF clinical trials and cardiovascular guidelines [10].

Heart failure presents, in most cases, as a chronic disease with a high burden of comorbidities, some of which seem to be sex-specific [6]. Whereas women are more likely to present comorbid hypertension, diabetes, hypothyroidism, obesity, and depression, men tend to have more peripheral vascular disease, ischemic heart disease and chronic obstructive pulmonary disease (COPD) [9,11,12]. The overall constellation of comorbidities surrounding HF may play an important role not only in the pathophysiology, but also in the treatment and prognosis of the disease [13].

Some differences in the response to treatment between men and women with HF might respond to specificities of their comorbidity pattern and of the pathophysiological origin of the disease. However, others might represent non-justified, gender-related differences [14]. On the other hand, due to the paucity of women recruited to HF trials, they show worse compliance with clinical practice guidelines, which are predominantly based on male-derived data. Women also receive lower average drug doses and show more adverse effects and worse prognoses in certain types of HF [4,9,15]. Similarly, care processes, resource use, and quality of care of HF patients may be different depending on their sex [16].

There is an unmet need of assessing whether sex differences in comorbidities of HF require specific management strategies. More studies focusing on women aimed at optimizing sex-specific therapy and, therefore, improving clinical outcomes in women, are needed [17]. Moreover, it has been suggested that HF may be a systemic, rather than a cardiac disorder, also involving other systems, such as the vascular, pulmonary, renal, gastrointestinal, and hepatic systems [18]. The identification of different comorbidity pathways in HF could provide evidence for individualized, person-centered care, targeting specific comorbidities and symptoms [19].

Combined efforts from different stakeholders (e.g., researchers, health authorities, and registry administrators) are required to adequately fill our knowledge gap on sex differences in HF [11]. In this context, the increasing availability for research of real-world, routinely collected health data obtained during the care process (e.g., electronic health records–EHRs) represents a unique opportunity to conduct clinical-epidemiological, large-scale population studies and provide us with real-world evidence on sex differences in HF.

The aim of this work was to describe the demographic, clinical, and health services’ use characteristics of a Spanish cohort of 17,516 men and women with HF based on real-world data, with a focus on the identification of sex differences.

## 2. Materials and Methods

We conducted a cross-sectional, observational study in the EpiChron Cohort, which links the information on demographics, diagnoses, drugs dispensed, and healthcare use rates of all public health system users in the Spanish region of Aragón. This cohort was conformed in 2011 including 1.25 million people at baseline (approximately 95% of the total inhabitants of the region). A description of the cohort and of the data sources was published elsewhere [20]. We selected for this study all prevalent patients with a diagnosis of HF in their primary or hospital EHRs on 1 January 2015 (i.e., 17,516 patients), and analyzed their demographic and clinical information corresponding to the year of study.

The Clinical Research Ethics Committee of Aragón (CEICA) approved the research protocol of this study (PI18/082), which complies with the Declaration of Helsinki. The CEICA waived the requirement to obtain informed consent from patients since all the information used was anonymized.

For each patient, we analyzed demographic data (i.e., age, sex, area of residence, acquisitive level, immigration status), all chronic condition diagnoses from primary and/or hospital care, all chronic medications dispensed, including those specifically used for the treatment of HF, monthly pharmacy expenditure, health services utilization (i.e., visits to primary care, specialties, hospital, and emergency department), self-reported data on drinking and smoking behaviors, and all-cause mortality. To determine the acquisitive level of each patient, we used the information regarding their type/degree of copayment of drugs covered by the public health system as a proxy variable to estimate patients’ individual annual income, which was divided into three categories as below 18,000 € (A), between 18,000 and 100,000 € (B), or above 100,000 €.

Diagnoses were extracted from the EHRs of both primary and hospital care levels, and included all active episodes between 1 January 2015 and 31 December 2015, even if a healthcare professional had recorded them before the initial date. Diseases were originally coded according to the International Classification of Primary Care (ICPC) or the International Classification of Diseases, Ninth Revision, Clinical Modification (ICD-9-CM), and subsequently grouped into 260 mutually exclusive Expanded Diagnostic Clusters (EDCs) using the Johns Hopkins ACG^®^ System (version 11.0, The Johns Hopkins University, Baltimore, MD, USA) [21]. We included every patient with the CAR05 EDC code, and considered for the analysis all 114 possible EDCs previously established as chronic by Salisbury et al. [22]. We defined multimorbidity as the presence of two or more EDCs from Salisbury’s list.

Chronic medications were defined as those with three or more dispensations over the 365-day follow-up period, using the Anatomical-Therapeutic-Chemical (ATC) classification system code at the third level. We defined polypharmacy as the simultaneous use of five or more drugs.

We performed a descriptive analysis of demographic, clinical, drug use, and health services use information of the population with HF based on their sex. The results were expressed as means, medians, and/or frequencies, accompanied by their corresponding standard deviations and/or interquartile ranges (IR). We compared age-adjusted means, medians, and frequencies using Student’s t-test, the Mann–Whitney U test, and chi-squared test, respectively. We also performed a multivariate Cox regression survival analysis to assess the effect of sex on 1-year mortality risk, after adjusting the hazard ratio (HR) by the age and number of comorbidities.

All statistical analyses were conducted using STATA (Version 12.0, StataCorp LLC, College Station, TX, USA). Statistical significance was set at *p* < 0.05.

## 3. Results

### 3.1. Overall Demographic and Clinical Characteristics

The majority of patients with HF in our cohort were women (57.4% vs. 42.6%, *p* < 0.001), who were, on average, older than men (Table 1). Although HF was more frequent in men in the population under 65 years, this condition was much more frequent in women over 65 years compared to their male counterparts, especially in the population over 85 years. No differences between men and women were shown in their immigrant status, area of residence, or acquisitive level. Women presented a slightly, although significant, higher body mass index (BMI) compared to men (30.3 vs. 29.6, *p* < 0.001), whereas men self-reported higher prevalence (%) of toxic habits, such as alcohol intake (26.2 vs. 3.0, *p* < 0.001) and smoking (12.4 vs. 3.5, *p* < 0.001).

Men and women with HF presented a similar disease burden, with 99 in 100 patients presenting multimorbidity, and 80% of them with six or more chronic conditions. The burden of drugs dispensed was also similar between sexes, with more than 80% of patients having polypharmacy. Men incurred, however, greater monthly pharmacy expenditure compared to women (1015 € vs. 821 €, *p* < 0.001).

### 3.2. Sex Differences in Comorbidity of HF

Both men and women showed a high comorbidity burden accompanying HF (Table 2). Hypertension (73%) and lipid metabolism disorders (45%) were the two most frequent chronic conditions in both men and women. Even after adjusting prevalence by age, some chronic conditions were clearly more frequent in women compared to men, such as (%) depression (29.0 vs. 13.0, *p* < 0.001), osteoporosis (26.2 vs. 4.5, *p* < 0.001), arthritis (42.0 vs. 27.5, *p* < 0.001), varicose veins (36.3 vs. 14.8, *p* < 0.001), hypothyroidism and other endocrine disorders (28.2 vs. 14.2, p < 0.001), and asthma (13.1 vs. 6.0, *p* < 0.001), among others. On the other hand, conditions such as COPD (32.3 vs. 14.0, *p* < 0.001), ischemic heart disease and acute myocardial infarction (39.9 vs. 22.1, *p* < 0.001), cardiac arrhythmia (44.5 vs. 37.0, *p* < 0.001), chronic renal failure (13.9 vs. 8.6, *p* < 0.001), gout (14.3 vs. 3.7, *p* < 0.001), behaviors problems (12.1 vs. 3.9, *p* < 0.001), cardiomyopathy (4.9 vs. 2.2, *p* < 0.001), and peripheral vascular disease (4.3 vs. 1.7, *p* < 0.001) were much more frequent in men. A much larger number of comorbidities showed sex differences in their prevalence (Table 2; Table S1), although not as clinically relevant as those mentioned above.

### 3.3. Pharmacology of HF

Drugs dispensed in the study population with HF are presented in Table 3. Sulphonamides diuretics were the most frequently dispensed medications for HF in both men (73.6%) and women (70.4%). The drugs recommended by clinical guidelines on HF were less frequently dispensed in women compared to men, such as beta-blocking agents (BB, 30.8% vs. 33.6%, *p* < 0.001), angiotensin-converting enzyme (ACE) inhibitors (25.8% vs. 33.1%, *p* < 0.001), and aldosterone antagonists (23.7% vs. 28.7%, *p* < 0.001), with the exception of angiotensin II receptor blockers (ARB, 23.8% vs. 21.0%, *p* < 0.001). The joint dispensing of ACE inhibitors, ARB, BB, and aldosterone antagonists was more frequent in men compared to women (8.4% vs. 6.1%, *p* < 0.001). Men also received more anti-aggregation (45.4% vs. 34.4%, *p* < 0.001) and anti-coagulation treatments with vitamin K antagonists (38.6% vs. 33.2%, *p* < 0.001) than women did.

Regarding the treatment of additional comorbidities, men were more frequently prescribed with drugs related to the treatment of respiratory diseases, such as inhaled adrenergic agents (32.3% vs. 24.0%, *p* < 0.001), anticholinergic agents (26.4% vs. 13.8%, *p* < 0.001), and corticosteroids for systemic use (17.3% vs. 15.1%, *p* < 0.001). On the other hand, non-steroidal anti-inflammatory and anti-rheumatic products (31.5% vs. 26.8%, *p* < 0.001), antidepressants (36.2% vs. 19.0%, *p* < 0.001), and thyroid hormones (12.8% vs. 4.6%, *p* < 0.001) were more frequently dispensed in women.

### 3.4. Health Services Use and Prognosis

The number of HF patients with at least one visit to the general practitioner (GP) was slightly higher in women than in men, although the number of annual visits (14.8 visits on average) was similar in both sexes (Table 4). Around 88% of patients with HF of both sexes visited nurses; however, women showed a more intensive use of this service (19.9 vs. 13.6 visits, *p* = 0.011).

On the other hand, a greater proportion of men than women used specialist care (79.0% vs. 70.6%, *p* < 0.001), and men had almost two more visits than women did. The number of different specialists visited was also greater in men compared to women (2.90 vs. 2.41, *p* < 0.001). Men also showed a greater hospitalization rate (47.0% vs. 38.1%, *p* < 0.001) and higher number of hospital admissions (0.87 vs. 0.65, *p* < 0.001), which were, on average, two days longer. The number of re-admissions to hospital during the 1-year follow-up period was also slightly higher in men compared to women. Regarding emergency care, men showed a greater utilization of this service compared to women (57.6% vs. 52.7%, *p* < 0.001), and more visits to the emergency room (1.32 vs. 1.15 visits, *p* < 0.001).

The Cox regression survival analysis revealed that women presented a 33% (HR 0.67, 95% CI 0.62–0.73) lower risk of 1-year mortality (*p* < 0.001) compared to men, after adjusting by age and number of comorbidities (Table 5).

## 4. Discussion

In this population-based study, we analyze the clinical characteristics of 17,516 patients with HF, and discuss the sex differences observed in their comorbidity, pharmacology, and healthcare utilization.

In our study, women contributed more than men to the total number of patients with HF. We therefore confirm the results of previous studies reporting a majority of women with HF in the general population. This would be due to the higher HF prevalence shown by women at advanced ages, both in the outpatient and hospital setting, that counteracts the initially higher HF incidence and mortality in men at earlier ages [7,8,23,24,25]. This was translated into a higher mean age of women in our study, similar to the results of previous studies that report a 2- to 3-year difference between both sexes [23,24,26]. Given that age seems to explain some of the differences reported between men and women with HF [8], we decided to adjust all our study outcomes by age.

One of the most questioned aspects of HF is whether women have a better prognosis than men. Our results seem to support the hypothesis of a greater survival rate of HF women compared to men. However, the effect on survival of sex varies depending on the cohort’s characteristics. In the BADAPIC (Base de Datos de Pacientes con Insuficiencia Cardiaca) study [9], conducted mainly in Spanish cardiology services, similar mortality rates were found for men and women. Nevertheless, patients were, on average, 15 years younger, and mostly men (71%). Conde–Martel et al. [23] reported, in internal medicine services, age-adjusted 1-year mortality rates of 28% and 25% in hospitalized HF men and women, respectively. In the Olmsted population study, 5-year mortality rates of 59% and 49% were found in ambulatory men and women [27,28]. In the I-PRESERVE study [29], in hospitalized patients with preserved left ventricular ejection fraction (LVEF), women presented a 20% lower risk of death for both cardiovascular and non-cardiovascular events. The MAGGIC meta-analysis [30], which included information on 41,949 patients, also showed a greater survival of women, suggesting that lower prevalence of ischemic heart disease, arrhythmias and symphatic activation, and better ventricular function are protective factors [26,31].

As expected, multimorbidity was the rule rather than the exception in our population, affecting up to 99% of patients of both sexes. The burden of diseases was considerably similar in men and women, who presented six or more chronic conditions in 80% of cases, and a median of nine conditions, similar to that found in other studies [6,24,25,32,33]. Carmona et al. [24] analyzed 198,670 patients, and more than 60% had five or more non-cardiac comorbidities. This multimorbidity might be justified by the advanced age of the population with HF, since one in four patients are older than 80 years and tend to present comorbid diseases [13,33,34]. It has to be highlighted that the high morbidity burden found in our study could be partially explained by the comprehensive list of chronic diseases analyzed [22].

Some comorbidities presented similar prevalence rates in both sexes. Other chronic conditions were, however, clearly specific to men or women. Men were more likely to present cardiovascular comorbidities, such as ischemic heart disease and acute myocardial infarction, cardiac arrhythmia, cardiomyopathy, peripheral vascular disease, COPD, and chronic renal failure, which is in line with the results of previous studies [9,12,23,26,32,35]. There are, however, controversies regarding the effect of sex on the prevalence of chronic renal failure and diabetes, with contradictory results in the literature [25,32,36,37,38,39]. The significant differences shown in COPD prevalence could be associated with higher levels of tobacco use in men [6,11,25], since toxic habits were clearly more frequent in men (although probably under-registered), as previously reported in the literature [8,11,26]. On the other hand, women were more likely to suffer conditions such as depression, osteoporosis, osteoarticular pathology, varicose veins, hypothyroidism, and asthma. Women also presented a slightly higher BMI, in agreement with various studies reporting more obesity in women, even tripling that reported in men in patients over 65 years [4,23,26,31].

The frequent multimorbidity of HF has been translated into more complex pharmacotherapy of the disease. In fact, over the past two decades, the taking of drugs has increased by more than six daily [34,35]. This trend is confirmed in our study, in which more than 80% of both men and women had five or more drugs dispensed. Current HF treatment guidelines are not sex-specific, and we therefore recommend the same management of the disease in men and women. However, women have been historically included in a smaller proportion (around 30%) than men in clinical trials, and their therapeutic management and prognosis have been less studied. All this leads to a lower prescription in women of the treatments recommended by clinical practice guidelines [12,31,40,41,42] and, for reasons not entirely clear, with lower average drug doses [9,43,44,45]. Although information on drug doses used and on the LVEF of our patients were not available in our cohort, we confirmed, in general, the lower prescription of the specific treatments for HF in women.

Males consumed more drugs for cardiovascular events than women did, which could be justified by the greater frequency of ischemic heart disease in men [6,41]. The greater combined use of ACE inhibitors/ARB, beta-blockers, and antialdosteronics could be justified by a higher prevalence of reduced LVEF in men [41]. In any case, the low use of ACE inhibitors/ARB and BB, which have been shown to improve the prognosis of the disease, as well as the high consumption of diuretics in clinically stable patients, is striking. Conde–Martel et al., among others, observed similar diuretics consumption in both sexes [23,26], whereas in other studies, greater use has been reported in men, despite women presenting with greater congestion [15,26,40]. The higher prevalence of ischemic heart disease and arrhythmias in men would justify the greater dispensation of anti-coagulation and anti-aggregation treatments, in line with the results of Dewan et al. [26], who also found a greater use of statins [6,41]. In other studies, greater side-effects related to ACE inhibitors have been reported for women, such as cough or angioedema [5,43,46,47]. In our sample, women had less arrhythmia and were also treated more frequently with digoxin than with beta-blockers, despite the greater association in women between use of digoxin and mortality and/or negative adverse effects [12,48,49]. It is worth highlighting the differences obtained regarding the BADAPIC study [9], conducted in Spain 10 years earlier. In contrast to our study, no sex differences were observed in the use of diuretics, digitalis, or anticoagulants. Beta-blockers and ACE inhibitors were dispensed more frequently in men in both studies, but these drugs were used much more frequently in the BADAPIC study (75% vs. 34% and 82% vs. 33% of men, respectively). These results could be partially explained by the greater presence of ischemia in BADAPIC patients.

Regarding healthcare use, it is worth noting the much greater use of nursing of women, probably due to their higher awareness of disease prevention. On the other hand, although the multimorbidity burden was similar in both sexes, men presented a greater consumption of hospital and specialized resources than women, which could be explained by the exacerbations and characteristics of their specific comorbidities, such as ischemic heart disease, arrhythmias, COPD, and chronic renal failure. In the study by Philibin et al. [16], it was shown that visits to specialties like cardiology were more frequent in men than in women. Also, in the study of the Olmsted population [27], men with HF had more annual hospital admissions than women (1.6 vs. 1.2 admissions). We did not study sex differences in the rate of readmission for HF, which was reported to be greater in women compared to men in the BADAPIC study, but in those patients referred to specialized HF clinics [9]. In any case, these data reflect the high fragmentation of care and resource consumption of patients with HF.

The role that the comorbidities and their treatment may play in the prognosis of HF is evidenced in our study by the high dispensation rates of drugs that could potentially decompensate a stable HF in both sexes, such as non-steroidal anti-inflammatory drugs, glucocorticoids, and beta-adrenergic inhalers. As we have seen, HF is, in most cases, accompanied by sex-specific multimorbidity and polypharmacy, which can potentially result in negative health outcomes due to drug–drug and drug–disease interactions if it is not correctly managed. This is the reason why person-centered, rather than disease-centered care is crucial in patients with this condition, in order to know the entire context of the patient and treat him/her appropriately to improve the prognosis of HF.

The main strength of the study is that we included almost all people with HF in the reference population from both primary and hospital care, so that men and women were equally represented. All the variables analyzed regarding comorbidity and healthcare use were extracted from patients’ EHRs. Therefore, this information should be more reliable and accurate than if it had been self-reported by patients, although some specific diseases could have potentially been over- or under-reported. In this regard, data in the EpiChron Cohort undergoes continuous quality control check-ups that ensure its accuracy and reliability for research purposes. Moreover, we comprehensively analyzed the comorbidities of HF by using an exhaustive list of chronic conditions, and not only the most prevalent or severe ones. Regarding HF pharmacology, the information was also reliable as it was obtained from pharmacy billing records; however, it represented the medications finally dispensed to the patient, and not all the drugs prescribed by health professionals.

One of the most important limitations of the study corresponds to the lack of data regarding the LVEF of the patients of the cohort, which was not retrievable from our information sources. We are aware that the impossibility of differentiating reduced from preserved LVEF HF does not allow us to analyze the effect of this relevant clinical variable on the assessment of sex differences in the management and prognosis of HF. Moreover, other relevant variables for the study, such as socioeconomic factors, were not available in the study cohort, whereas other ones, such as lifestyle habits, were unavailable (e.g., diet, physical activity) or probably underreported (e.g., smoking and drinking behaviors). Another major limitation is the cross-sectional nature of the study. We characterized all prevalent cases of HF at a given time frame, without taking into account the time elapsed since the diagnosis of the disease nor the chronological appearance of comorbidities over time.

## 5. Conclusions

This cohort study, based on real-world data of all patients with a diagnosis of HF of a public health system, confirms clinically relevant differences between men and women regarding their comorbidity profiles, pharmacological treatment plans, patterns of use of health care services, and prognosis. These results, that should shortly be replicated to be confirmed in another contexts, may help to orientate the design of specific strategies for secondary prevention of chronic diseases associated to HF, for the early detection of specific comorbidities, and for the development of sex-specific clinical guidelines for HF patients to reduce negative health outcomes and increase the quality of life of this population group.

## Figures and Tables

**Table 1:** Demographic and clinical description of the study population with heart failure based on their sex.

Characteristics	Men	Women	*p* Value
**N (%)**	7454 (42.6%)	10,062 (57.4%)	<0.001
**Age**			
**Median (IR ^a^)**	80 (15)	83 (11)	<0.001
**Age interval, years (n, %)**			<0.001
≤44	281 (1.6%)	223 (1.3%)	
45–64	886 (5.1%)	572 (3.3%)	
65–84	4146 (23.7%)	4837 (27.6%)	
≥85	2141 (12.2%)	4430 (25.3%)	
**Immigrant (n, %)**	83 (1.1%)	122 (1.2%)	0.595
**Residence, rural (n, %)**	3575 (48.0%)	4444 (44.2%)	0.981
**Acquisitive level ^b^ (n, %)**			0.060
A	474 (6.4%)	732 (7.3%)	
B	6917 (92.8%)	9245 (91.9%)	
C	63 (0.8%)	84 (0.8%)	
**BMI ^c^ (median, IR)**	29.6 (6.04)	30.3 (7.83)	<0.001
**Toxic habits ^d^ (n, %)**			
Alcohol consumption, yes	1954 (26.2%)	300 (3.0%)	<0.001
Smoking, yes	924 (12.4%)	349 (3.5%)	<0.001
**Chronic diseases ^e^**			
**Median (IR)**	9 (6)	9 (6)	0.910
**Number of diseases (n, %)**			0.307
1	84 (1.1%)	95 (0.9%)	
2	153 (2.1%)	186 (1.8%)	
3	262 (3.5%)	337 (3.3%)	
4	423 (5.7%)	544 (5.4%)	
5	569 (7.6%)	713 (7.1%)	
≥6	5962 (80%)	8187 (81.4%)	
**Multimorbidity ^f^ (n, %)**	7370 (98.9%)	9967 (99.1%)	0.234
**Drugs dispensed ^g^**			
**Median (IR)**	8 (5)	8 (5)	0.385
**Number of drugs (n, %)**			0.258
0–1	227 (3.7%)	348 (4.1%)	
2–4	707 (11.5%)	1002 (11.9%)	
≥5	5209 (84.8%)	7043 (83.9%)	
**Monthly pharmacy expenditure, € (median, IR) ^h^**	1015 (−1237)	821 (−1111)	<0.001

^a^ Interquartile range; ^b^ Proxy variable of acquisitive level of patients according to their annual individual income: below 18,000 € (A), between 18,000 and 100,000 € (B), or above 100,000 € (C); ^c^ Body mass index; ^d^ Self-reported by the patient and retrieved from patient’s general descriptors; ^e^ From a list of 114 chronic conditions including HF, age-adjusted means are presented; ^f^ Defined as the presence of two or more chronic conditions, HF included; ^g^ Using the Anatomical-Therapeutic-Chemical classification code at the third level, age-adjusted means are presented; ^h^ Calculated with the retail price of each drug.

**Table 2:** Differences in the comorbidity profile of men (*n* = 7454) and women (*n* = 10,062) with heart failure. Age-adjusted prevalence of chronic comorbidities with a mean prevalence higher than 5% is presented.

Chronic Condition ^a^	Men (n, %)	Women (n, %)	*p* Value
Hypertension	5292 (71.0)	7476 (74.3)	<0.001
Disorders of lipid metabolism	3280 (44.0)	4629 (46.0)	0.009
Cardiac arrhythmia	3317 (44.5)	3723 (37.0)	<0.001
Arthritis	2050 (27.5)	4226 (42.0)	<0.001
Diabetes	2661 (35.7)	3210 (31.9)	<0.001
Hematologic disorders, other	2177 (29.2)	2666 (26.5)	<0.001
Cataract, aphakia	1968 (26.4)	2848 (28.3)	0.008
Varicose veins of lower extremities	1103 (14.8)	3653 (36.3)	<0.001
Emphysema, chronic bronchitis, COPD ^b^	2408 (32.3)	1409 (14.0)	<0.001
Cardiovascular disorders, other	1811 (24.3)	2194 (21.8)	<0.001
Obesity	1498 (20.1)	2616 (26.0)	<0.001
Musculoskeletal disorders, other	1319 (17.7)	2485 (24.7)	<0.001
Depression	969 (13.0)	2918 (29.0)	<0.001
Ischemic heart disease (excluding AMI ^c^)	1804 (24.2)	1560 (15.5)	<0.001
Iron deficiency, other deficiency anemias	1394 (18.7)	1992 (19.8)	0.055
Musculoskeletal signs and symptoms	1290 (17.3)	1922 (19.1)	0.002
Cerebrovascular disease	1304 (17.5)	1690 (16.8)	0.216
Cardiac valve disorders	1275 (17.1)	1721 (17.1)	0.999
Surgical aftercare	1357 (18.2)	1469 (14.6)	<0.001
Sleep disorders of nonorganic origin	1118 (15.0)	1741 (17.3)	<0.001
Osteoporosis	335 (4.5)	2636 (26.2)	<0.001
Respiratory signs and symptoms	1230 (16.5)	1378 (13.7)	<0.001
Respiratory disorders, other	1275 (17.1)	1238 (12.3)	<0.001
Dermatitis and eczema	1006 (13.5)	1248 (12.4)	0.036
Chronic ulcer of the skin	894 (12.0)	1378 (13.7)	<0.001
Deafness, hearing loss	932 (12.5)	1298 (12.9)	0.521
Glaucoma	894 (12.0)	1248 (12.4)	0.412
Chronic renal failure	1036 (13.9)	865 (8.6)	<0.001
Acute myocardial infarction	1170 (15.7)	664 (6.6)	<0.001
Other endocrine disorders	596 (8.0)	1409 (14.0)	<0.001
Hypothyroidism	462 (6.2)	1489 (14.8)	<0.001
Neurologic disorders, other	671 (9.0)	1127 (11.2)	<0.001
Asthma	447 (6.0)	1318 (13.1)	<0.001
Gout	1066 (14.3)	372 (3.7)	<0.001
Cardiovascular signs and symptoms	678 (9.1)	855 (8.5)	0.176
Behaviour problems	902 (12.1)	392 (3.9)	<0.001
Blindness	574 (7.7)	815 (8.1)	0.304
Thrombophlebitis	455 (6.1)	885 (8.8)	<0.001
Diverticular disease of colon	499 (6.7)	735 (7.3)	0.117
Low back pain	514 (6.9)	674 (6.7)	0.515
Neurologic signs and symptoms	432 (5.8)	724 (7.2)	<0.001
Acute renal failure	574 (7.7)	463 (4.6)	<0.001
Conjunctivitis, keratitis	410 (5.5)	644 (6.4)	0.013
Allergic rhinitis	447 (6.0)	574 (5.7)	0.302
Psychological signs and symptoms	321 (4.3)	624 (6.2)	<0.001
Substance use	239 (3.2)	694 (6.9)	<0.001

^a^ From a list of 114 chronic conditions, listed in descending order of mean prevalence; ^b^ Chronic obstructive pulmonary disease; ^c^ Acute myocardial infarction.

**Table 3:** Differences in drug dispensation between men (*n* = 7454) and women (*n* = 10,062) with heart failure. Age-adjusted dispensation rates of drugs of interest are shown.

Medication	Men (n, %)	Women (n, %)	*p* Value
**Anticoagulants**			
Vitamin K antagonists	2877 (38.6)	3341 (33.2)	<0.001
Direct thrombin inhibitors	142 (1.9)	161 (1.6)	0.237
Direct factor Xa inhibitors	298 (4.0)	392 (3.9)	0.689
**Antiplatelet**			
Platelet aggregation inhibitors excl. heparin	3384 (45.4)	3461 (34.4)	<0.001
**Diuretics**			
Thiazides, plain	127 (1.7)	171 (1.7)	0.867
Sulphonamides, plain	5486 (73.6)	7084 (70.4)	<0.001
Aldosterone antagonists	2139 (28.7)	2385 (23.7)	<0.001
**Beta-blockers (BB)**			
BB, non-selective	52 (0.7)	111 (1.1)	0.004
BB, selective	2505 (33.6)	3099 (30.8)	<0.001
Alpha and beta blocking agents	716 (9.6)	694 (6.9)	<0.001
**Calcium antagonists**			
Dihydropyridine derivatives	1200 (16.1)	1419 (14.1)	<0.001
Phenylalkylamine derivatives	52 (0.7)	121 (1.2)	0.003
Benzothiazepine derivatives	790 (10.6)	1078 (10.7)	0.829
**Digitalis glycosides**	1334 (17.9)	1962 (19.5)	0.006
**ACE ^a^ inhibitors/ARB ^b^**			
ACE inhibitors, plain	2467 (33.1)	2596 (25.8)	<0.001
ARB, plain	1565 (21.0)	2395 (23.8)	<0.001
**Lipid modifying agents, plain**	3541 (47.5)	4246 (42.2)	<0.001
**Anti-inflammatory and antirheumatic products, non-steroids**	1998 (26.8)	3170 (31.5)	<0.001
**Glucocorticoids**	1290 (17.3)	1519 (15.1)	<0.001
**Thyroid hormones**	343 (4.6)	1288 (12.8)	<0.001
**Antidepressants**	1416 (19.0)	3642 (36.2)	<0.001
**Inhaled therapy**			
Adrenergics, inhalants	2408 (32.3)	2415 (24.0)	<0.001
Anticholinergics	1968 (26.4)	1389 (13.8)	<0.001

^a^ Angiotensin converting enzyme; ^b^ Angiotensin II receptor blockers.

**Table 4:** Differences in the pattern of utilization of health services in men (*n* = 7454) and women (*n* = 10,062) with heart failure during the year of study. Age-adjusted healthcare use rates are shown.

Health Services Use	Men	Women	*p* Value
**Primary care**			
Patients with at least 1 visit to GP ^a^ (n, %)	6917 (92.8)	9478 (94.2)	<0.001
Number of visits to GP (mean, sd ^b^)	14.7 (12.3)	14.9 (12.1)	0.343
Patients with at least 1 visit to nursing (n, %)	6552 (87.9)	8855 (88.0)	0.838
Number of visits to nursing (mean, sd)	13.6 (18.4)	19.9 (16.9)	0.011
**Specialist care**			
Patients with at least 1 visit to any specialist (n, %)	5889 (79.0)	7104 (70.6)	<0.001
Number of visits to specialties (mean, sd)	8.51 (10.5)	6.83 (9.1)	<0.001
Number of different specialists visited (n, %)			<0.001
1	1014 (13.6)	1499 (14.9)	
2	1140 (15.3)	1469 (14.6)	
3	1051 (14.1)	1258 (12.5)	
4	850 (11.4)	986 (9.8)	
5	678 (9.1)	704 (7.0)	
≥6	1155 (15.5)	1197 (11.9)	
Mean (sd)	2.90 (2.48)	2.41 (2.40)	<0.001
**Hospital care**			
Patients with at least 1 hospital admission (n, %)	3503 (47.0)	3834 (38.1)	<0.001
Number of hospital admissions (mean, sd)	0.87 (1.31)	0.65 (1.10)	<0.001
Length of stay, days (mean, sd)	8.25 (15.5)	6.39 (13.22)	<0.001
Number of readmissions (mean, sd)	0.19 (0.60)	0.16 (0.52)	<0.001
**Emergency care**			
Patients with at least 1 visit to emergency room (n, %)	4294 (57.6)	5303 (52.7)	<0.001
Number of visits (mean, sd)	1.32 (1.93)	1.15 (1.70)	<0.001

^a^ General Practitioner; ^b^ standard deviation.

**Table 5:** Cox regression survival analysis to assess the risk of 1-year mortality in men and women with heart failure based on sex, age, and number of comorbidities.

Variable	Hazard Ratio ^a^	95% CI ^b^	*p* Value
**Sex** (Reference: men)	0.672	0.621–0.726	<0.001
**Age**^c^ (years)	1.077	1.071–1.083	<0.001
**Number of comorbidities** ^d^	1.057	1.047–1.067	<0.001

^a^ Adjusted by the rest of variables; ^b^ 95% confidence interval; ^c^ Continuous variable; ^d^ Continuous variable, excluding heart failure.

## Supplementary Materials

The following are available online at https://www.mdpi.com/1660-4601/17/6/2136/s1, Table S1: Differences in the entire comorbidity profile of men (*n* = 7454) and women (*n* = 10,062) with heart failure.

## References

[1] Tan L.B., Williams S.G., Tan D.K.H., Cohen-Solal A. (2010). So many definitions of heart failure: Are they all universally valid? A critical appraisal. Expert Rev. Cardiovasc..

[2] Ponikowski P., Anker S.D., AlHabib K.F., Cowie M.R., Force T.L., Hu S., Jaarsma T., Krum H., Rastogi V., Rohde L.E. (2014). Heart failure: Preventing disease and death worldwide. Esc. Heart Fail..

[3] Savarese G., D’Amario D. (2018). Sex differences in heart failure. Advances in Experimental Medicine and Biology.

[4] Lam C.S.P., Arnott C., Beale A.L., Chandramouli C., Hilfiker-Kleiner D., Kaye D.M., Ky B., Santema B.T., Sliwa K., Voors A.A. (2019). Sex differences in heart failure. Eur. Heart J..

[5] Eisenberg E., Di Palo K.E., Piña I.L. (2018). Sex differences in heart failure. Clin. Cardiol..

[6] Gimeno-Miguel A., Gracia Gutiérrez A., Poblador-Plou B., Coscollar-Santaliestra C., Pérez-Calvo J.I., Divo M.J., Calderón-Larrañaga A., Prados-Torres A., Ruiz-Laiglesia F.J. (2019). Multimorbidity patterns in patients with heart failure: An observational Spanish study based on electronic health records. BMJ Open.

[7] Leiro M.G.C., Martín M.J.P. (2006). Heart failure. Are women different?. Rev. Esp. Cardiol..

[8] Strömberg A., Mårtensson J. (2003). Gender differences in patients with heart failure. Eur. J. Cardiovasc. Nurs..

[9] Jiménez-Navarro M.F., Ramírez-MArrero M.A., Anguita-Sánchez M., Castillo J.C., For the BADAPIC Investigators (2010). Influence of gender on long-term prognosis of patients with chronic heart failure seen in heart failure clinics. Clin. Cardiol..

[10] Sardar M.R., Badri M., Prince C.T., Seltzer J., Kowey P.R. (2014). Underrepresentation of women, elderly patients, and racial minorities in the randomized trials used for cardiovascular guidelines. JAMA Intern. Med..

[11] Hopper I., Kotecha D., Chin K.L., Mentz R.J., Von Lueder T.G. (2016). Comorbidities in heart failure: Are there gender differences?. Curr. Heart Fail. Rep..

[12] Lin F., Greenberg B. (2020). Considering the gender gap in heart failure. Eur. J. Heart Fail..

[13] Van Deursen V.M., Urso R., Laroche C., Damman K., Dahlström U., Tavazzi L., Maggioni A.P., Voors A.A. (2014). Co-morbidities in patients with heart failure: An analysis of the European Heart Failure Pilot Survey. Eur. J. Heart Fail..

[14] Baumhäkel M., Müller U., Böhm M. (2009). Influence of gender of physicians and patients on guideline-recommended treatment of chronic heart failure in a cross-sectional study. Eur. J. Heart Fail..

[15] Kajimoto K., Sato N. (2020). Sex differences in New York Heart Association Functional Classification and survival in acute heart failure patients with preserved or reduced ejection fraction. Can. J. Cardiol..

[16] Philbin E.F., DiSalvo T.G. (1998). Influence of race and gender on care process, resource use, and hospital-based outcomes in congestive heart failure. Am. J. Cardiol..

[17] Franconi F., Carru C., Malorni W., Vella S., Mercuro G. (2011). The Effect of sex/gender on cardiovascular pharmacology. Curr. Pharm. Des..

[18] Davison B., Cotter G. (2015). Why is heart failure so important in the 21st century?. Eur. J. Heart Fail..

[19] Lawson C.A., Solis-Trapala I., Dahlstrom U., Mamas M., Jaarsma T., Kadam U.T., Stromberg A. (2018). Comorbidity health pathways in heart failure patients: A sequences-of-regressions analysis using cross-sectional data from 10,575 patients in the Swedish Heart Failure Registry. PLoS Med..

[20] Prados-Torres A., Poblador-Plou B., Gimeno-Miguel A., Calderón-Larrañaga A., Poncel-Falcó A., Gimeno-Feliú L.A., González-Rubio F., Laguna-Berna C., Marta-Moreno J., Clerencia-Sierra M. (2018). Cohort Profile: The epidemiology of chronic diseases and multimorbidity. The EpiChron Cohort Study. Int. J. Epidemiol..

[21] The Johns Hopkins University ACG® System. https://www.hopkinsacg.org/.

[22] Salisbury C., Johnson L., Purdy S., Valderas J.M., Montgomery A.A. (2011). Epidemiology and impact of multimorbidity in primary care: A retrospective cohort study. Br. J. Gen. Pr..

[23] Conde-Martel A., Arkuch M.E., Formiga F., Manzano-Espinosa L., Aramburu-Bodas O., González-Franco Á, Dávila-Ramos M.F., Suárez-Pedreira I., Herrero-Domingo A., Montero-Pérez-Barquero M. (2015). Gender related differences in clinical profile and outcome of patients with heart failure. Results of the RICA Registry. Rev. Clin. Esp..

[24] Carmona M., García-Olmos L.M., Alberquilla A., Muñoz A., García-Sagredo P., Somolinos R., Pascual-Carrasco M., Salvador C.H., Monteagudo J.L. (2011). Heart failure in the family practice: A study of the prevalence and comorbidity. Fam. Pr..

[25] Formiga F., Ferrer A., Sanz H., Marengoni A., Alburquerque J., Pujol R., Octabaix Study Members (2013). Patterns of comorbidity and multimorbidity in the oldest old: The Octabaix study. Eur. J. Intern. Med..

[26] Dewan P., Rorth R., Raparelli V., Campbell R.T., Shen L., Jhund P.S., Petrie M.C., Anand I.S., Carson P.E., Desai A.S. (2019). Sex-related differences in heart failure with preserved ejection fraction. Circ. Heart Fail..

[27] Gerber Y., Weston S.A., Redfield M.M., Chamberlain A.M., Manemann S.M., Jiang R., Killian J.M., Roger V.L. (2015). A contemporary appraisal of the heart failure epidemic in Olmsted, Minnesota, 2000 to 2010. JAMA Intern. Med..

[28] Levy D., Kenchaiah S., Larson M.G., Benjamin E.J., Kupka M.J., Ho K.K., Murabito J.M., Vasan R.S. (2002). Long-term trends in the incidence of and survival with heart failure. N. Engl. J. Med..

[29] Lam C.S., Carson P.E., Anand I.S., Rector T.S., Kuskowski M., Komajda M., McKelvie R.S., McMurray J.J., Zile M.R., Massie B.M. (2012). Sex differences in clinical characteristics and outcomes in elderly patients with heart failure and preserved ejection fraction: The Irbesartan in Heart Failure with Preserved Ejection Fraction (I-PRESERVE) trial. Circ. Heart Fail..

[30] Martinez-Sellés M., Doughty R.N., Poppe K., Whalley G.A., Earle N., Tribouilloy C., McMurray J.J., Swedberg K., Kober L., Berry C. (2012). Meta-Analysis Global Group in Chronic Heart Failure (Mggic). Gender and survival in patients with heart failure: Interactions with diabetes and aetiology. Results fromthe MAGGIC individual patient meta-analysis. Eur. J. Heart Fail..

[31] Bozkurt B., Khalaf S. (2017). Heart failure in women. Methodist Debakey Cardiovasc. J..

[32] Chamberlain A.M., St Sauver J.L., Gerber Y., Manemann S.M., Boyd C.M., Dunlay S.M., Roca W.A., Finney Rutten L.J., Jiang R., Weston S.A. (2015). Multimorbidity in heart failure: A community perspective. Am. J. Med..

[33] Braunstein J.B., Anderson G.F., Gerstenblith G., Weller W., Niefeld M., Herbert R., Wu A.W. (2003). Noncardiac comorbidity increases preventable hospitalizations and mortality among Medicare beneficiaries with chronic heart failure. J. Am. Coll. Cardiol..

[34] Butrous H., Hummei S.L. (2016). Heart failue in older adults. Can. J. Cardiol..

[35] Wong C.Y., Chaudhry S.I., Desai M.M., Krumholz S.M. (2011). Trends in comorbidity, disability, and polypharmacy in heart failure. Am. J. Med..

[36] Meyer S., Van der Meer P., Massie B., O’Connor C.M., Metra M., Ponikowski P., Teerlink J.R., Cotter G., Davison B.A., Cleland J.G. (2013). Sex-specific acute heart failure phenotypes and outcomes from PROTECT. Eur. J. Heart Fail..

[37] Klempfner R., Koifman E., Goldenberg I., Hamdan A., Tofler G.H., Kopel E. (2014). The Israel Nationwide Heart Failure Survey: Sex differences in early and late mortality for hospitalized heart failure patients. J. Card. Fail..

[38] Shah A.N., Mentz R.J., Gheorghiade M., Kwasny M.J., Fought A.J., Zannad F., Swedberg K., Maggioni A.P., Konstam M.A. (2012). Gender does not affect postdischarge outcomes in patients hospitalized for worsening heart failure with reduced ejection fraction (from the Efficacy of Vasopressin Antagonism in Heart Failure Outcome Study withTolvaptan (EVEREST) Trial). Am. J. Cardiol..

[39] Hsich E.M., Grau-Sepulveda M.V., Hernandez A.F., Peterson E.D., Schwamm L.H., Bhatt D.L., Fonarow G.C. (2012). Sex differences in in-hospital mortality in acute descompensated heart failure with reduced and preserved ejection fraction. Am. Heart J..

[40] Cai S., Gong I.Y., Gale C.P., Yan A.T. (2020). Sex-specific differences in New York Heart Association Classification and outcomes of descompensated heart failure. Can. J. Cardiol..

[41] Lainscak M., Milinkovi I., Polovina M., Crespo-Leiro M.G., Lund L.H., Anker S.D., Laroche C., Ferrari R., Coats A.J.S., McDonagh T. (2020). Sex-and age-related differences in the management and outcomes of chronic heart failure: An analysis of patients from the ESC HFA EORP heart failure long-term registry. Eur. J. Heart Fail..

[42] Norberg H., Pranic V., Bergdahl E., Lindmark K. (2020). Differences in medical treatment and clinical characteristics between men and women with heart failure- a single-centre multivariable analysis. Eur. J. Clin. Pharm..

[43] Marra A., Salzano A., Arcopinto M., Piccioli L., Raparelli V. (2018). The impact of gender in cardiovascular medicine: Lessons fron the gender/sex-issue in heart failure. Monaldi Arch. Chest Dis..

[44] Regitz-Zagrosek V. (2006). Therapeutic implications of the gender-specific aspects of cardiovascular disease. Nat. Rev. Drug Discov..

[45] Rosano G.M., Lewis B., Agewall S., Wassmann S., Vitale C., Schmidt H., Drexel H., Patak A., Torp-Pedersen C., Kjeldsen K.P. (2015). Gender differences in the effect of cardiovascular drugs: A position document of the working group on pharmacology and drug therapy of the ESC. Eur. Heart J..

[46] Bots S.H., Groepenhoff F., Eikendal A., Tannenbaum C., Rochon P.A., Regitz-Zagrosek V., Miller V.M., Day D., Asselbergs F.W., Den Ruijter H.M. (2019). Adverse drug reactions to guideline-recommended heart failure drugs in women: A systematic review of the literature. Jacc. Heart Fail..

[47] Yancy C.W., Jessup M., Bozkurt B., Butler J., Casey D.E., Colvin M.M., Drazner M.H., Filippatos G., Fonarow G.C., Givertz M.M. (2016). Correction to: 2016 ACC/AHA/HFSA Focused Update on new pharmacological therapy fo heart failure: An update of the 2013 ACCF/AHA Guideline for the management of heart failure: A report of the American College of Cardiology Foundation/American Heart Association task force on clinical practice guidelines and the Heart Failure Society of America. Circulation.

[48] Rathore S.S., Wan Y., Krumholz H.M. (2002). Sex-based differences in the effect of digoxin for the treatment of heart failure. N. Engl. J. Med..

[49] Adams F.K., Patterson J.H., Gattis W.A., O’Connor C.M., Lee C.R., Schwartz T.A., Gheorghiade M. (2005). Relationship of serum digoxin concentration to mortality and morbidity in women in the digitalis investigation group trial: A retrospective analysis. J. Am. Coll. Cardiol..

